# Detecting Seasonal Flow Pathways in Road Structures Using Tracer Tests and ERT

**DOI:** 10.1007/s11270-018-4008-6

**Published:** 2018-10-26

**Authors:** Hedi Rasul, Robert Earon, Bo Olofsson

**Affiliations:** 1 Division of Land and Water Resources Engineering, Royal Institute of Technology (KTH), 100 44 Stockholm, Sweden; 2 Department of Civil Engineering, Faculty of Engineering, Koya University, KOY45, Koya, Kurdistan Region – F.R. Iraq

**Keywords:** ERT, Tracer test, Flow pathways, Road, Hydrology

## Abstract

Roads and traffic can be a source of water-bound pollutants, which can percolate through the unsaturated zone to groundwater. Deicing salt is widely used on roads in northern Europe during winter and is usually applied at a time when the temperature is below zero and the soil is partly frozen. Understanding the mechanism by which water-bound pollutants such as deicing salt are transferred from roads to groundwater is highly important for groundwater protection, environmental sustainability and road maintenance. Electrical resistivity tomography (ERT) can be used for tracing the infiltration of deicing salt in different seasons, including the frozen period, as a step towards identifying pollutant infiltration pathways. In this study, a tracer-ERT monitoring method and analytical process was developed and evaluated for use in investigating and demonstrating deicing salt infiltration pathways in road structures in different seasons and weather conditions. The method involves using dissolved sodium chloride as a tracer and monitoring its infiltration using a multi-electrode array system. The tracer tests were performed at the same location in different seasons over a 1-year period. The results indicated high seasonal variation in percolation pattern and flow velocity, with large decreases in December (winter), most likely due to preferential flow paths within the road shoulder. These findings can be applied to other water-soluble pollutants that move from the road surface to groundwater.

## Introduction

The main source of groundwater is percolation of precipitation through the unsaturated zone. Percolation pathways can differ depending on soil type and soil structures. Some recharge areas are in close proximity to road networks, which can be a source of pollutants transferring to groundwater. In Sweden and many other countries, it is common to use deicing salt on roads during winter. In most cases when deicing salt is applied, the temperature is around or below zero and the soil is sometimes frozen. Understanding the mechanism by which deicing salt spreads from roads to groundwater is critical for sustainability and groundwater protection.

Part of the deicing salt applied spreads by infiltration from the road shoulder into the soil and percolates to groundwater. Roads have been identified as a source of pollution for soil and water in a number of studies (Hoffman 1985; Howard and Haynes 1993; Blomqvist and Johansson 1999; Thunqvist 2000; Turer et al. 2001; Lindström 2005; Lundmark and Olofsson 2007; Earon et al. 2012). The amount of deicing salt applied varies depending on the weather, type of road and traffic conditions (Blomqvist et al. 2011). Deicing salt is available in large amounts compared with other pollutants from roads (Earon et al. 2012). Thus, it is one of the main pollutants in countries in cold regions and, as it has limited reactivity and usually follows the percolating water, it can function as an excellent tracer. Deicing salt can spread from road to surrounding environment by infiltration, runoff and splash (Lundmark and Olofsson 2007), increasing the chloride content in surface water and groundwater. Olofsson and Sandström (1998) investigated deep wells within 500 m from main roads in Sweden and concluded that 50% of their chloride content originated from deicing salt. Meriano et al. (2009) investigated the impact of chloride and concluded that it is often greatest after short thaw events during winter, when it can reach 2000 mg/L. Those authors thus recommend monitoring of groundwater contamination. Studies modelling the flow paths in the road structure (Apul et al. 2007) and through road shoulders (Hansson et al. 2005; Olofsson et al. 2017) have demonstrated the importance of the latter for the process, especially as modern road shoulders are highly susceptible to infiltration (Paulsson 2008; Dawson 2009). Deposition of deicing salt occurs mainly within 10 m of the roadside (Astebol et al. 1996; McBean and Al-Nassri 1987), although some investigations show that airborne particles after splash can spread up to 100 m and more (Blomqvist and Johansson 1999). Other investigations suggest that the solutes also transport horizontally within the capillary fringe (Persson et al. 2015). Investigations of flow pathways into the road structure are highly problematic on trafficked roads. Non-destructive methods of investigation for use on and near roads are therefore required, in order to preserve the integrity of the road and avoid disrupting traffic. Geophysical methods are fast and non-destructive, and are frequently applied in environmental investigations (Johansson and Dahlin 1996; French et al. 2002; Jackson et al. 2002; Olofsson et al. 2005; Leroux and Dahlin 2006; Lundmark and Olofsson 2007; Rosqvist et al. 2007; Chambers et al. 2008; Earon et al. 2012; Dailey et al. 2014). Olofsson et al. (2017) performed tracer tests using sodium chloride and geophysical measurements on different road types in Sweden. However, previous studies have all been conducted during the warm summer season and previous investigations of frost problems in the road structure have focused on the top layer (Hermansson 2002; Hermansson et al. 2009). To our knowledge, no previous study has examined seasonal variations in flow pathways into road shoulders and percolation through the road structure, especially during the frozen period.

Using electrical resistivity tomography (ERT) to monitor and investigate the process of infiltration in different seasons, in particular the frozen period, can be a step forward in understanding the pathways of deicing salt. Studies using ERT have been carried out previously (Gheith and Schwartz 1998; French and Van der Zee 1999; French et al. 1999, 2002; Aaltonen 2001; Aaltonen and Olofsson 2002; Cassiani et al. 2006), but not directly related to roads. The aim of the present study was to investigate the flow pathways in the road material beneath a typical road surface during different seasons, using tracer test experiments and monitoring with a non-destructive ERT method. The impact of frozen soil on flow pathways was also investigated. Moreover, salt concentration changes in the road material were estimated from resistivity changes, in order to investigate retardation of salt within the road material during the study period.

## Methods

### Site Description

**Fig. 1:**
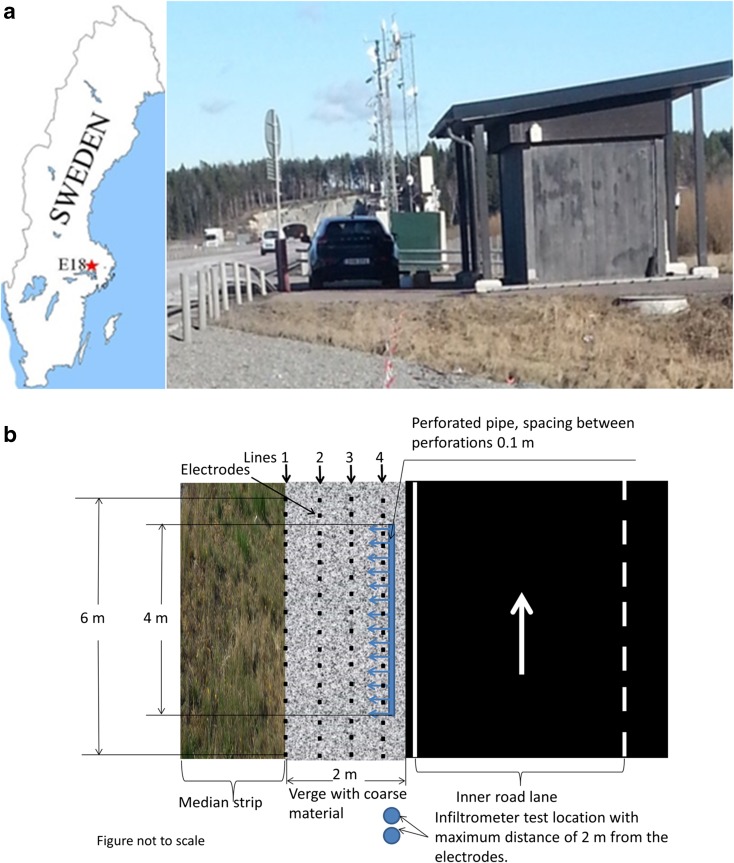
**a** Location of Testsite E18 in Sweden and its appearance. **b** Electrical resistivity tomography (ERT) devices in the road shoulders for the tracer test

The field work for this study was conducted at a test station on European motorway E18 north-west of Stockholm, central Sweden. The test station is located between the cities of Västerås and Enköping (Fig. 1a). The official name for the station is Testsite E18, and it is the first environmental road research station in Sweden (Testsite E18 2015). The geological materials under the site are soils comprised mostly of glacial and post-glacial clay sediments, covering glacial till and bedrock (Earon et al. 2012). According to measurements taken at the station, the groundwater level is between 5 and 6.25 m below the surface. At this test station there are many different sensors which collect meteorological, hydrological and traffic data simultaneously. In addition to these permanent sensors, a temporary set of resistivity sensors was installed for tracer test measurements on the road shoulders in the present study (Fig. 1b).

### Data Collection and Processing

#### Resistivity Survey and Tracer Test

Four tracer tests were carried out at different times in order to study the variation under wet and dry conditions during spring (April), summer (August) and winter (December). Sodium chloride dissolved in water was used as a tracer. Resistivity progress was monitored by a multi-electrode array system (ABEM_LS) that consisted of four lines of 16 electrodes (4 × 16), with 0.5 m between the lines and 0.4 m between the electrodes. The tracer was injected beside the line 4, close to inner lane at a distance of 40 cm. The perforated pipe used was 4 m long and had a spacing between perforations of 10 cm. A pole-dipole protocol was used, as described by Olofsson and Lundmark (2010).

Measurements carried out before adding the tracer were used as background levels. The tracer, which consisted of 50 L water containing 30,000 mg/L NaCl, was then added via a perforated 4-m-long pipe, one perforation per each 0.1 m, simulating deicing salt runoff from the surface of the road pavement towards the shoulder. In this study, the tracer concentration used was higher than in Singha and Gorelick (2005), since we assumed that the background conductivity will be higher in chloride content due to previous salt applications from which no detailed information was available. The concentration of 30,000 mg/L (30 g/L) was an estimated value close to the applied salt. NaCl was used as the tracer since it is rather safe for health and the environment (Tilly et al. 1999). All electrodes were placed on the gravel shoulder, starting at 0.3–0.5 m from the edge of the paved surface, and the tracer was injected using a perforated pipe which was placed at 0.3 m from the paved surface with the direction of the perforations towards the resistivity lines (Fig. 1b). Electrical resistivity measurements were made every 30 min, with each series of measurements comprising 736 measurements taking roughly 25–35 min to collect. In total, four to six series of measurements were made during each test, with a measurement time lapse of 0.0, 0.5, 1.0, 2.0 and 2.5 h depending on the contact between electrodes and ground surface. In some cases, e.g. during the August measurements, the surface ground was very dry and the contact between electrodes and ground surface was very poor, which eliminated all except four measurement series. The last measurement series in April was taken 22 h after adding the tracer and the last measurement in December was taken after 5 h after adding the tracer. Longer time lapse measurements were taken only for the tracer test in April as it was the only case where the tracer had not penetrated fully within the first time lapse. The data collected were then subjected to inverse modelling using Res2DInv and Res3Dinv software (Loke 2016). The results were presented as pseudo 3D plots produced by Voxler, using the analytical outputs from Res2DInv to create an xyz plot. Due to high inversion error from Res3DInv outputs, only inversion from Res2DInv was used in producing the pseudo 3D plots. Inversion error for 2D resistivity was less than 5% in all four lines. The inverted data were corrected for temperature variations. Matlab-B and OriginLab (OriginLab 2015) software were used for further calculations.

#### Corrections Due to Temperature

Resistivity data were corrected to 25 °C standard temperature, based on Keller and Frischknecht (1966) and Brunet et al. (2010). The actual temperature (*T*) was recorded by the sensors in the road material available at the test site. The correction equation used was

1 $$ {\rho}_{25}={\rho}_T+\alpha\ {\rho}_T\ \left(T-25\right) $$

where *ρ*_25_ is the electrical resistivity corrected to a temperature of 25 °C, *ρ*_*T*_ is the electrical resistivity at temperature *T* and α is a coefficient obtained empirically and often equal to 0.025 °C^−1^ (Brunet et al. 2010).

#### Estimation of Chloride Concentration From Electrical Resistivity

In order to estimate the change in salt concentration from the change in electrical resistivity, Archie’s law (Archie 1942) was used. It is a petrophysical equation relating salinity and electrical resistivity that is determined empirically for sand samples (porosity ranging from 10 to 40%) as

2 $$ {R}_o=F{R}_f $$

where *R*_*o*_ is the resistivity of saturated sand, *R*_*f*_ is the resistivity of fluid or brine in the pores and *F* is a formation factor defined as

3 $$ F={n}^{-m} $$

where *n* is the porosity and *m* is an empirical parameter related to degree of pore connectivity (cementation exponent). The parameter m is less than 2 for most poorly cemented materials (Keller and Frischknecht 1966; Singha and Gorelick 2005). In the present study, its value was set to 2 in the calculations due to the low clay content in road material. Porosity was estimated to be 21–31 vol.%, based on the Meinzer (1923) value for a sandy and gravelly mix, which is the most reasonable approximation to the sub-grade material and other road material at the study site. In the calculations, we have used a porosity value of 28% in order to follow Singha and Gorelick (2005). Based on the literature (Singha and Gorelick 2005, 2006), the change in apparent conductivity was used to estimate the chloride concentration (Singha and Gorelick 2005) as

4 $$ {\Delta  C}_{cl-}=3.19\times F\times \Delta  {\sigma}_o $$

where *∆C*_*cl*−_ is the estimated change in chloride concentration calculated from electrical resistivity in mg/L and *∆σ*_*o*_ is the apparent conductivity change in mS/m. Multi-level sampler data for constructing the correlation between Eq. 1 and chloride concentration changes were used, based on Singha and Gorelick (2005). The results were obtained by the following procedure: conductivity data were corrected for measured soil temperature, using Eq. 1. Seasonal conductivity changes from the first measurements, in April 2015, were then calculated using Eq. 4. Finally, for each measurement, the sum of all changes in the chloride concentration was calculated.

#### Other Measured Data at the Test Site

Infiltration tests were carried out using a double-ring infiltrometer with rather small inner diameter (14.4 cm) due to the limited width of the shoulder. On each of the test days, two to four infiltration tests were carried out on the shoulder near the tracer test location. During winter, due to frozen ground, the infiltrometer ring did not penetrate the gravel material well, so clay was used as a seal between the infiltrometer and the ground. The test was repeated a few times with some results discarded due to leakage of water beneath the infiltrometer. All infiltration tests were made at the same location beside the tracer test location, within 2 m from the outer electrodes (Fig. 1b).

Weather parameters, such as temperature, precipitation and moisture before and during the tests, were also considered. Precipitation data were downloaded from the Swedish Meteorological and Hydrological Institute (SMHI) website (SMHI 2015). Precipitation data were used to compare and discuss the results from the different test days. Air and soil temperature data are continuously measured at the test site and are available online, updated every 10 min. The soil temperature sensors at the test site register the temperature from the asphalt layer down to a depth of 2 m beneath the surface.

## Results

### Temperature and Precipitation Measurements

**Fig. 2:**
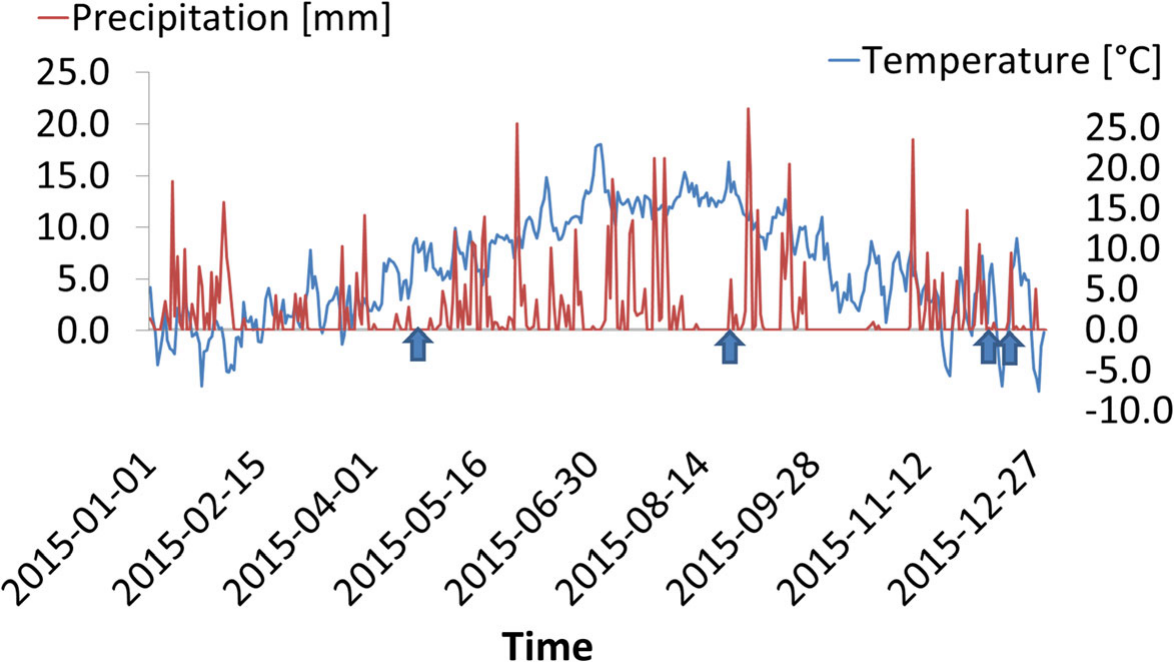
Precipitation and air temperature during the study period. Tracer tests are indicated by arrows

Air temperature and precipitation during the tracer tests are presented in Fig. 2. As the precipitation curve shows, on 23 April, there was no precipitation, but on 27 August, 0.4 mm of rain was recorded. On 8 December, the soil was covered by a thin layer of snow, whereas the bare soil was frozen on 16 December. The air temperature curve reached almost 20 °C in August, whereas the December measurements were taken during a cold period with temperatures below zero.

**Fig. 3:**
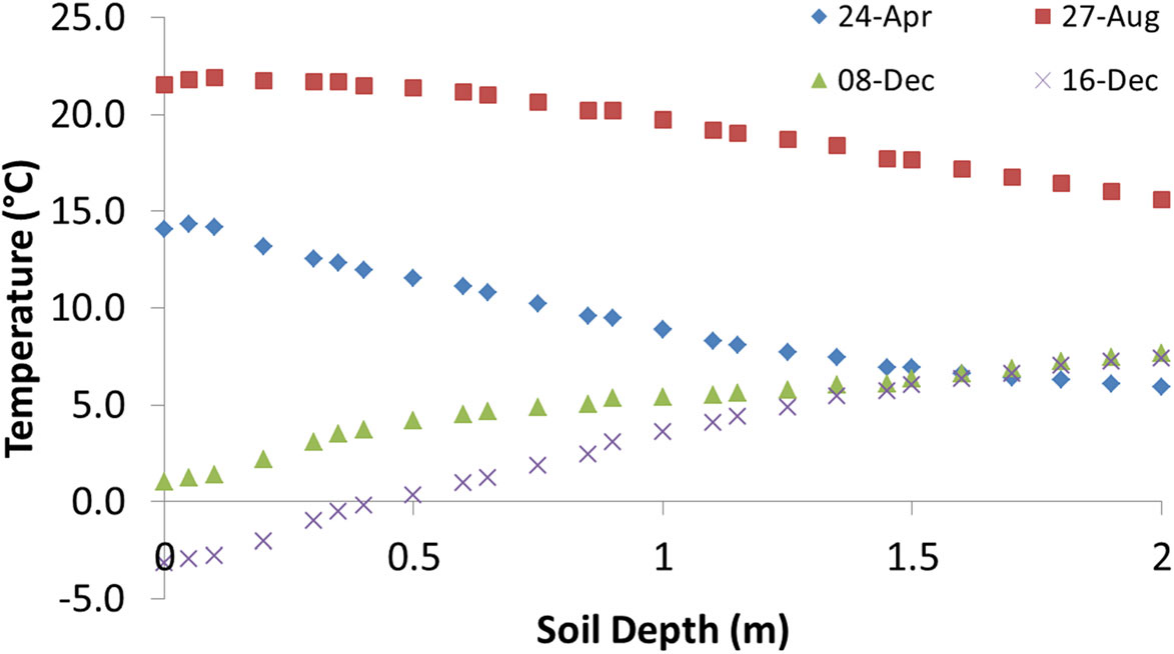
Temperature in the road material down to 2 m below the road surface on the tracer test days

The measured soil temperature at different depths during the tracer tests is presented in Fig. 3. As the temperature curves indicate, there was great variation in surface temperature in different seasons, but at 2-m depth, the variation was very low. Data from August showing higher temperatures were compared with data from the other seasons.

### Infiltration Measurements

**Fig. 4:**
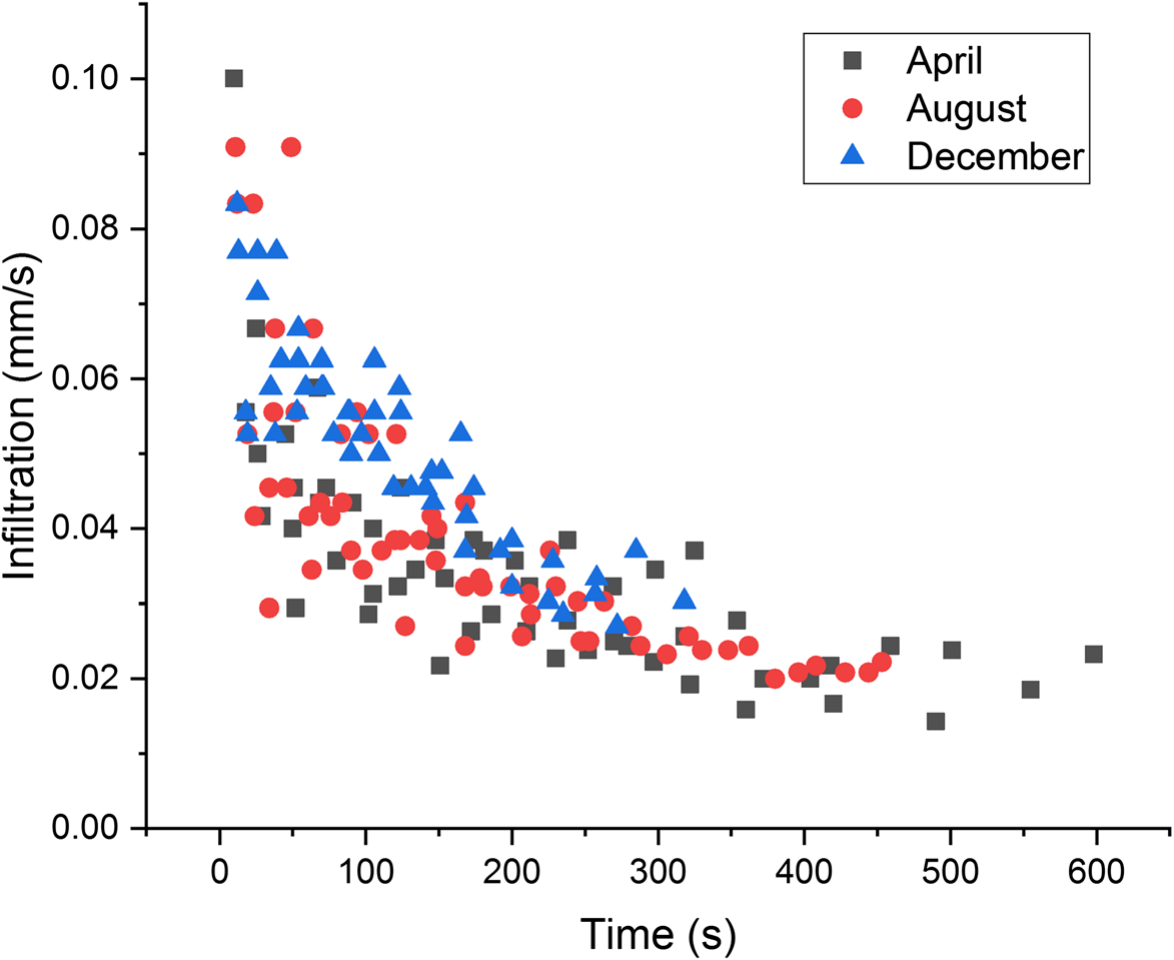
Results obtained using the double-ring infiltration test (all measurements)

The results from the infiltration measurements are presented in Fig. 4. In April infiltration was about 0.02 mm/s, which was much lower than at the measurements in August and December. In August, infiltration was faster after the first set of measurements, since the initially dry road material impeded the infiltration process. In December, the infiltration process was similar to that in August, but due to some frozen pores and ground, the flow did not reach stable conditions. Lateral flow occurred within the top few centimetres of the soil. In general, the initial infiltration was high in all seasons and it varied at different locations depending on varying grain sizes in the shoulder. Within less than 10 min, the average infiltration at most locations was between 0.02 and 0.04 mm/s, which is low compared with the time needed for 50 L of tracer to infiltrate over an area of approximately 4 m^2^. During the tracer tests, the time needed for the entire volume of tracer to infiltrate was less than 3 min. Based on the tracer test, the average infiltration was estimated to be 0.07 mm/s, which was higher than determined in the double-ring infiltrometer test.

### Resistivity Changes in 1D for Each Resistivity Line

**Fig. 5:**
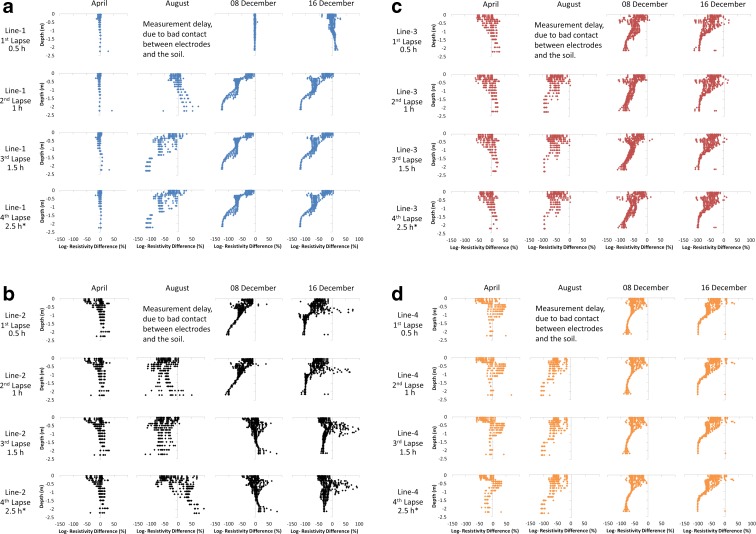
Percentage logarithmic change in resistivity in **a** line 1, **b** line 2, **c** line 3 and **d** line 4, with time lapses of 0.5, 1.0, 1.5 and 2.5 h. Asterisk indicates that the last time lapse was not fixed and was 22 h in the April tracer test

Resistivity changes as a function of depth down to 2 m due to addition of tracers were compared against the initial resistivity values for each measurement line (Fig. 5). The percentage of resistivity changes in comparison to the background measurement were calculated for each resistivity line and plotted (Fig. 5). Since the tracer contains chloride, the resistivity in the soil was expected to decrease, but some increases in resistivity were modelled at greater depths, which were assumed to be inversion artefacts (Rasul et al. 2018). The reductions in resistivity were the primary focus in the current study. The reduction in resistivity was found to be greater in lines 3 and 4, as the tracer was added beside line 4 (close to the paved surface, whilst line 1 was located 2 m from the paved surface, close to the inner ditch; see Fig. 1). The depth of infiltration in April, observed in the measurements after 22 h, was roughly 2 m. In the first three measurement series in April, only the resistivity in the top layer decreased, which indicates that tracer was retarded in the top layers and gradually percolated further down. After 22 h, the resistivity modelled in line 4 decreased down to 2 m below the surface, which was the maximum depth modelled by the array.

In measurements taken in April, the resistivity changes were very small and mostly uniformly distributed, whilst in August, there were some highly negative changes but, in comparison with December, the negative resistivity changes were less clustered. Measurements taken in December showed that resistivity changes were greater in magnitude than those in the previous months and that the spatial distribution of these resistivity changes was different, with more clustered sharp negative changes per each line. Measurements taken in the warmer months showed both positive and negative resistivity changes at all depths, indicating erratic but highly non-uniform spatial behaviour. Measurements taken in the colder months showed a very strong decrease in resistivity for all measurement lines except the first time lapse for line 1. This could indicate the presence of preferential flow paths, with measurements still unaffected by the tracer (Fig. 5).

### Resistivity Changes in Combined 2D

**Fig. 6:**
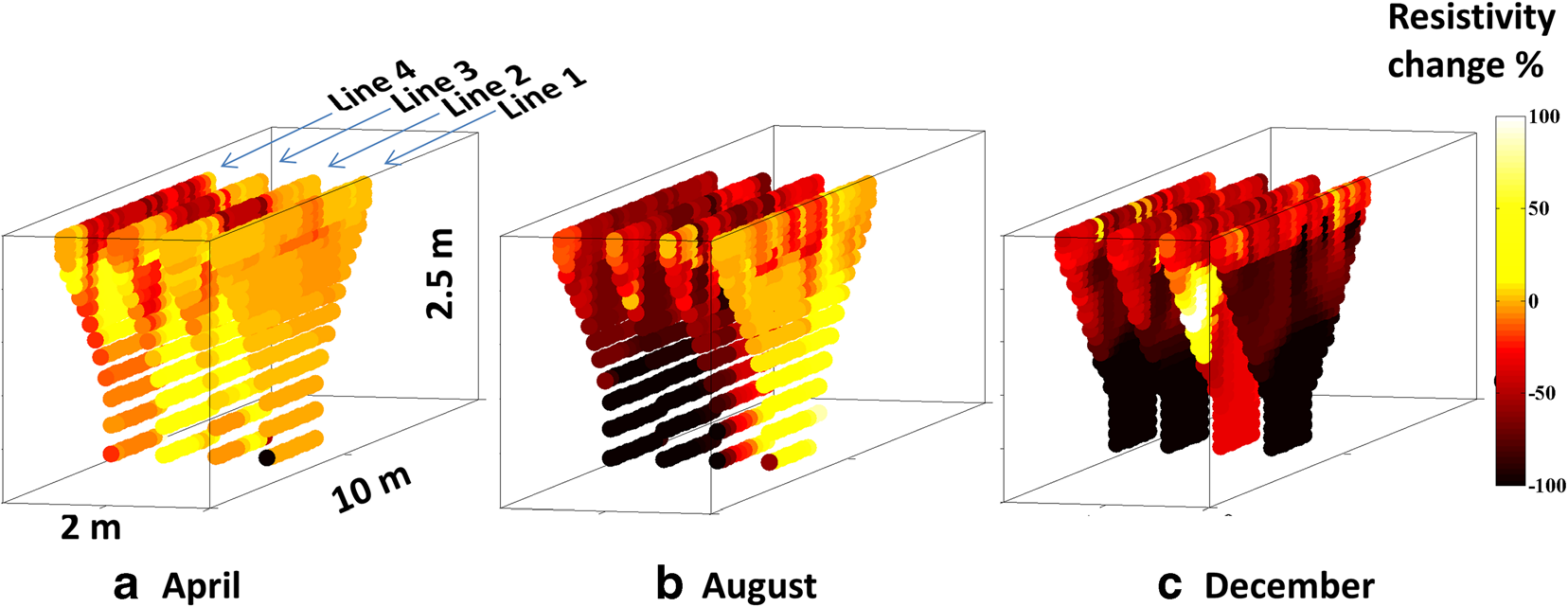
Percentage change in resistivity change for all lines in combined 2D presentations after the second time lapse (1 h) of the tracer tests in **a** April, **b** August and **c** December

Highly non-uniform spatial behaviour were observed for measurements made in April compared with measurements made in August and on 16 December, as shown after the second time lapse (=1 h) in Fig. 6. In April, the change in resistivity occurred in a significantly smaller volume than in August and December. Only the resistivity lines close to the road were affected. The weather and road material conditions during the test in April were wet and the soil was saturated, so when using a fixed volume of tracer (50 L of water with 30,000 mg/L NaCl), it was only possible to detect reduced resistivity within the top layers. However, in the measurements taken in August (Fig. 6b) and December (Fig. 6c), resistivity changes were observed in the entire depth (to 2 m), which indicates fast percolation, but in August, the spread of resistivity decrease was less within the superficial layers and all lines were not affected. This is similar to the findings based on the 1D results.

### Resistivity Changes in Pseudo 3D

**Fig. 7:**
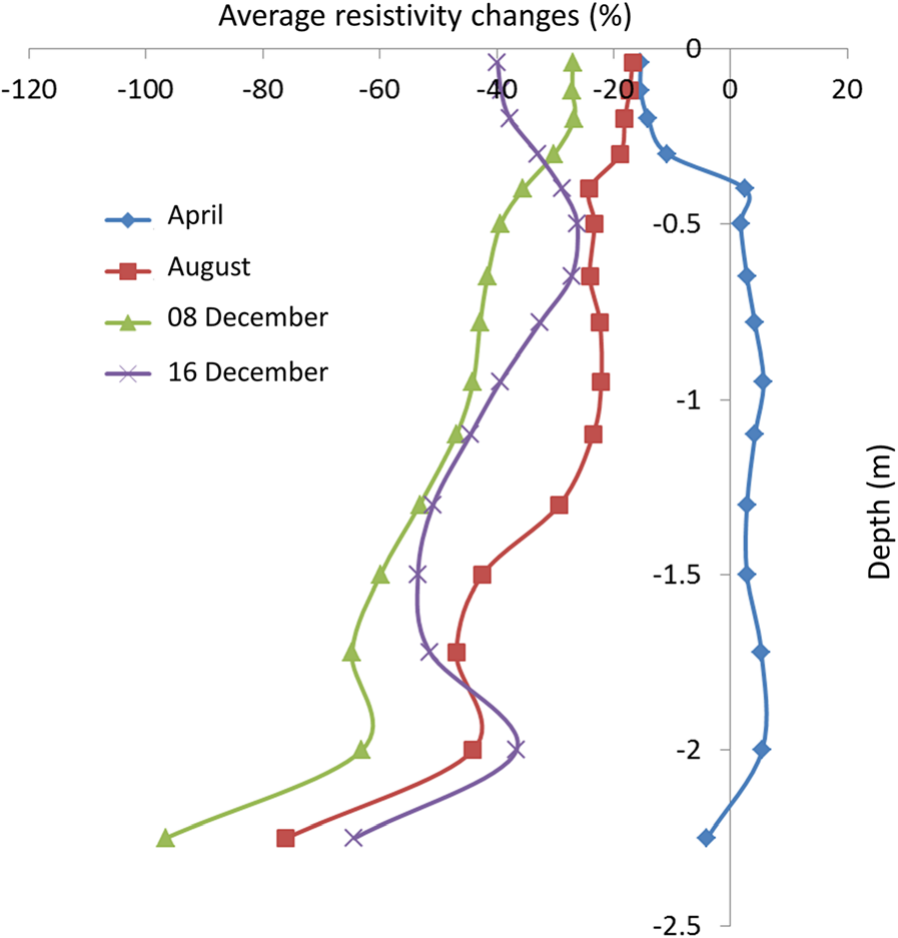
Resistivity changes plotted in pseudo 3D after the first time lapse, with contours representing a 22% resistivity reduction in **a** April, **b** August and **c** December

**Fig. 8:**
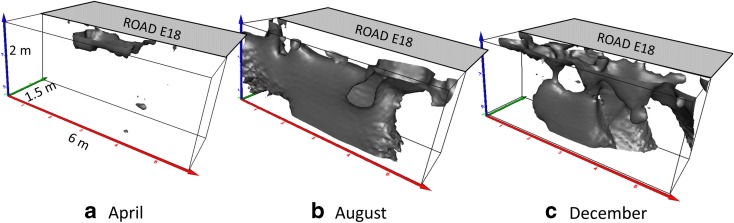
Percentage average resistivity change with depth in different seasons

The soil volumes affected by resistivity changes from the infiltrated tracer are presented in Fig. 7, with contour lines showing 22% resistivity reduction after the first time lapse of the tracer tests in April, August and December. In April, resistivity changes occurred only near the surface; in August, the changes affected most of the soil volume; and on 16 December, clear preferential flow was detected. This was seen in all time lapse analyses. Plotting average resistivity for all measurements as a function of depth (Fig. 8) revealed that the change in resistivity in April mostly occurred in the upper part of the road, down to 40 cm, which indicated that the tracer flow was retarded, probably due to the high degree of water saturation in the soil. In August, the ground was rather dry and the tracer caused a rapid change in resistivity down to 1.5 m. The effect was quite similar on 8 December, except that the decrease in resistivity was more pronounced than during previous months. The changes in resistivity with depth on 16 December, when the top layer was frozen, revealed that the tracer penetrated downwards but more locally, indicating preferential flow. Eventually, the tracer managed to percolate down to a depth of more than 2 m.

During the test period, there was no precipitation in the form of snow, according to the SMHI database. However, on 8 December, we observed 1–2 cm of snow or frost on the road shoulder during the tracer test measurements. This means that there was no snow ploughing and no high accumulation of snow on the road shoulders, which would have prevented infiltration. During the test year (2015), year-round infiltration through the shoulder material occurred rather than stormwater runoff through the drain system.

### Chloride Concentration Estimations

**Fig. 9:**
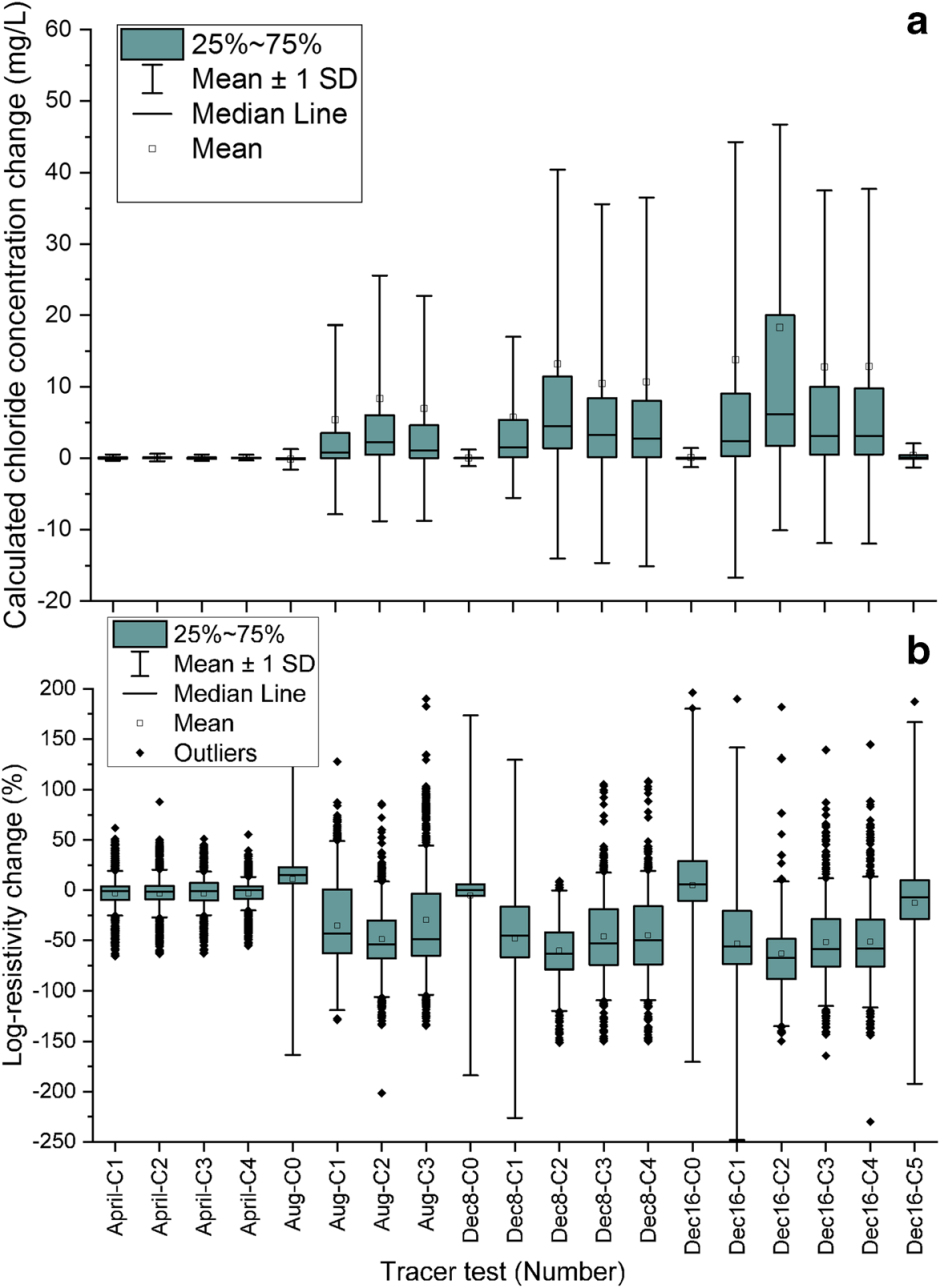
**a** Calculated chloride concentration changes for all measurements compared with the background measurement in April. **b** Percentage change in resistivity for all measurements compared with the background level (i.e. Aug-C0, Dec8-C0 and Dec16-C0). C1 to C5 represent the time lapse measurement series

Calculated change in chloride concentration compared with the first background level was plotted to identify general trends in the chloride concentration (Fig. 9). Tracer tests Aug-C0, Dec8-C0 and Dec16-C0 in the diagram represent the background measurements for tracer tests in August and on 8 December and 16 December, respectively. As can be seen from Fig. 9a, the overall chloride concentrations in the road layers increased over time within the test year. However, the chloride concentration during the tracer test in April did not differ greatly from the background level due to retardation of the tracer. The reference measurement series in April showed very small changes in chloride concentration compared with the other background measurements in August and December. The last measurement in the study year (Dec16–05) also showed a reduction in the chloride concentration. After adding the tracer, the chloride concentration initially increased but started to decrease from the third measurement series, indicating that the number of cells affected by the tracer was reduced. Some of the tracer was probably lost, since it percolated down to the groundwater. The corresponding percentage changes in resistivity are shown in Fig. 9b. The percentage negative resistivity change increased and average resistivity changes in each of the tracer tests decreased during the first two measurement series compared with the background measurements. In the last measurement series in each tracer test, the resistivity changes decreased again due to loss of tracer downwards.

## Discussion

Understanding the mechanism by which deicing salt spreads from roads to the surrounding environment is of vital importance for sustainability and groundwater protection. The results generally show the flow pathways and trend of water and tracer spread in the road shoulders in different seasons. Using electrical resistivity tomography method in monitoring tracer and general chloride concentration changes was a very good method to address such results without causing any disturbances and excavations in the road shoulders. By this research, it is possible to advance the current knowledge and understanding of the water and pollutant spread in the road layers. However, the method comprised a range of uncertainties. The actual soil temperature at the tracer test location may have been slightly different from the measured soil temperature on the site, because the soil temperature sensor was located under the road lane near the shoulder at a more protected site and there were no sensors directly under the shoulder which is more exposed. The main discrepancy was observed on 8 December, when the soil temperature sensor showed temperatures above zero but frozen moisture was observed covering the coarse shoulder material during the tracer test, so the soil temperature was probably below zero. In winter and spring, the groundwater level was high and the temperature was around 6 °C at a depth of 2 m. In August, the temperature was higher than 15 °C at 2-m depth, which clearly shows that on the summer test day, the ground was dry and the temperature measurements could reach high values. The decrease in resistivity due to the tracer was more pronounced in August and December than in April when a thin plume remained in the top layers, leading to more difficulty in its precise detection by resistivity methods (Whiteley and Jewell 1992; Aaltonen 2001). Rapid penetration of the tracer at the top 2 m has previously been observed by Slater et al. (1997). Rapid downward infiltration can cause environmental consequences after road accidents (Olofsson et al. 2017). The results indicated highly heterogeneous flow patterns due to heterogeneous porous media. The results were varying during different seasons; the overall picture of flow paths would not have been achieved by taking the tracer test only during one season. The retardation of the tracer on the surface layers in April may have been due to the wet ground and also dilution of the tracer. The chloride estimation based on the resistivity measurements was based on several assumptions from previous studies (Meinzer 1923; Keller and Frischknecht 1966; Singha and Gorelick 2005, 2006; Brunet et al. 2010), and no laboratory studies of the parameters were used. This caused an increase of the uncertainty in the chloride estimation but the results were not used for detailed calculation of chloride changes. In order to decrease the impact of uncertainties, the general changes of the chloride content between different tracer tests have been compared and analysed without going into more detail by separating them to different lines or levels.

In this study, no background conductivity of the groundwater was measured since there were no test bore holes near the tracer test location. In addition, the background values of the chloride content were assumed to be raised due to previous salt applications for which there is no accurate historical data. The concentration of 30,000 mg/L was an estimated value close to assumed applied salt. On each tracer test day, at least two infiltration tests were collected. During winter, due to frozen ground, the infiltrometer ring did not penetrate the gravel material well, so clay was used to seal the joint between the infiltrometer and the ground. A few times the test was repeated due to leakage of water beneath the infiltrometer. Longer time lapses, up to 22 h after injection, were taken only for the tracer test in April because it was the only case where the tracer was not penetrated fully within the first time lapse.

Since no road material samples were collected, the porosity and other site parameters were assumed based on the literature and Earon et al. (2012). Porosity ranges were within the range of Archie’s law application. The measured apparent resistivity was corrected due to temperature based on Keller and Frischknecht (1966). Then the rest of the parameters were estimated based on literature values (Meinzer 1923; Brunet et al. 2010; Singha and Gorelick 2005, 2006). Since collection of ERT data is an indirect method which do not give specific chloride concentrations at specific depths, we recommend that a full-scale test should be carried out where the actual chloride concentration of the road material should be analysed after tracer injection by soil sampling and chemical analyses at different depths. Such studies are, however, very difficult to perform at a main road in operation since it is costly and destructive. Conventional drilling and soil sampling might not be a good option since it cannot give representative values in heterogeneous media, but combining both methods by taking samples based on the ERT results might give a better estimation in chloride concentration changes near the main pathways. Even though the assumptions cause several uncertainties, these uncertainties will be the same within all the different tracer tests and the actual study only pinpoints general trends and variations of the infiltration and sub-surface flow patterns between the different tracer tests within various seasons. The general trend of chloride content in the soil raise by time and some chloride probably are preserved in the soil but are dissolved again during next tracer injection.

Regarding the fact that the measured resistivity sometimes seems to increase to some high positive values, it is likely that it is the result of an inversion artefact during the modelling procedure as previous investigations faced similar results (Rasul et al. 2018). The decreases in resistivity were the primary focus of this research since injection of a chloride tracer should lead to significant negative changes of resistivity if the chloride concentration of the injected liquid is higher than the already existing chloride concentration in the pore voids. For measuring the real chloride concentration, we need to collect soil samples.

## Conclusions

Using a non-destructive ERT method to monitor tracer movements proved to be a good approach for analysing and monitoring flow pathways in road layers. During tracer tests in spring with wet ground conditions, infiltration and percolation occurred to significantly smaller depth than in dry conditions. Most of the tracer remained on or within the top layer of the shoulder, resulting in increased stormwater runoff. When the ground was dry, as in the tests in August, there was little stormwater runoff, but the percolation was deep and instant, forming a more homogeneous network of pathways. Finger flow was more apparent during tests in winter on frozen ground. Despite these preferential flow paths developing in frozen ground, some chloride was still retained in the road surface layers. Therefore, for better estimation and calibration of the chloride concentration, road material should be sampled at different depths for chemical analyses with taking into account the heterogeneity of the road material, since ERT data do not show pollutants and concentrations in specific locations. The experiment was focused on investigating how flow paths and transport within the unbound shoulder varies during different seasons. The tests show that the transport pathways and flow characteristics vary significant. A large portion of deicing salt was infiltrated in the shoulder and was not present at the surface as runoff except during the wet spring season. There are many remaining questions, for example the portion of chloride accumulating in the road body and how much chloride reaches the groundwater. In addition, more research is needed regarding other types of road pollutants spread through road shoulders.
